# Land‐Use Shapes Soil Fungal Communities and Functional Guilds in Tropical Forest Ecosystems

**DOI:** 10.1002/pei3.70193

**Published:** 2026-08-04

**Authors:** Damilola Olanipon, Margaux Boeraeve, Adams Iyiola, Munir Wahab, Hans Jacquemyn

**Affiliations:** ^1^ Department of Biological Sciences Afe Babalola University Ado Ekiti Nigeria; ^2^ Centre for Functional and Evolutionary Ecology, (CEFE) Univ Montpellier, CNRS, EPHE Montpellier France; ^3^ Biology Department KU Leuven Heverlee Belgium; ^4^ Department of Biology UAntwerpen Antwerpen Belgium; ^5^ Department of Fisheries and Aquatic Resources Management College of Agriculture, Osun State University Ejigbo Osun State Nigeria; ^6^ Department of Wildlife and Ecotourism Management, College of Agriculture Osun State University Ejigbo Osun State Nigeria

**Keywords:** agroforestry, community composition, conservation, deforestation, land‐use change, soil health, tropical forest

## Abstract

Land‐use change is a major driver of biodiversity loss and soil degradation in tropical ecosystems, yet its impacts on soil fungal communities, particularly arbuscular mycorrhizal fungi (AMF), remain poorly understood. Here, we investigated how fungal diversity and community composition differed between three land use types (cropland, disturbed, and undisturbed forest vegetation) in two forest reserves in southwestern Nigeria. We hypothesized that intact forests harbor the most diverse fungal communities, while croplands would be dominated by a less diverse community of more disturbance‐tolerant fungi. Using high‐throughput sequencing of the Internal Transcribed Spacer (ITS) and Small Subunit (SSU) regions, we detected 9240 fungal operational taxonomic units (OTUs) and 738 AMF amplicon sequence variants. Fungal communities were dominated by *Ascomycota*, while AMF communities were primarily represented by the genus *Glomus*, followed by *Paraglomus*, *Diversispora*, *Acaulospora*, and *Claroideoglomus*. Contrary to expectations, we found no significant differences in AMF communities between land‐use types (*F* = 1.1, *p* = 0.351). Total fungal, saprotrophic and plant pathogenic fungal communities, on the other hand, clearly differed between land‐use types. Furthermore, indicator species analysis identified a greater number of unique fungal OTUs in undisturbed forests, suggesting that while generalists persist, specialist fungal taxa are lost in disturbed sites. Overall, our findings indicate that agroforestry, where perennial crops maintain host root continuity, may buffer mycorrhizal fungal assemblages against the negative effects of deforestation, but at the same time highlight the importance of conserving multiple forest habitats to protect site‐specific belowground biodiversity.

## Graphical abstract

Land‐use change is a major cause of biodiversity loss and soil degradation in tropical regions. The role of soil fungi, including arbuscular mycorrhizal fungi (AMF), though critical, is often overlooked. AMF communities across cropland, disturbed forest, and undisturbed forest vegetation in southwestern Nigeria were relatively resilient to land‐use change, likely due to agroforestry practices. Saprotrophic and plant pathogenic fungi respond strongly to land‐use change, with specialist species lost from degraded sites. Agroforestry can partially buffer the impacts of deforestation on soil ecosystems and safeguard unique soil biodiversity.

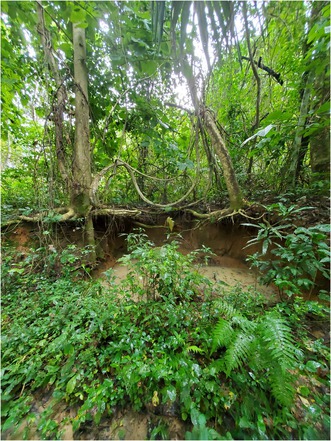

## 1 Introduction

Soils harbor the highest microbial diversity on Earth (Banerjee and Van Der Heijden 2023), and this vast belowground diversity is crucial for essential ecosystem functions, including nutrient cycling, soil aggregation, and plant productivity (Rillig et al. 2015; Manral et al. 2023). Soil microbes also contribute to carbon sequestration and climate regulation (Treseder and Holden 2013; Bargali et al. 2019), thereby linking belowground processes to global biogeochemical cycles. Microbial communities therefore play an essential role in maintaining soil and plant health, and supporting ecosystem restoration (Coban et al. 2022; Xiao et al. 2023).

Among soil microorganisms, fungi are key drivers of organic matter decomposition, nutrient mineralization, and overall ecosystem functioning (Tedersoo et al. 2014; Van Der Heijden et al. 2008; Zhou et al. 2025). The composition and diversity of soil fungi are largely influenced by vegetation cover, litter quality, and soil physicochemical conditions (Li et al. 2023). Natural forests typically support diverse fungal communities that regulate nutrient retention, carbon storage, and biogeochemical cycling, whereas agricultural soils often contain reduced fungal richness due to intensive tillage, fertilizer use, and limited organic inputs (Spurgeon et al. 2013; Song et al. 2015; Wang et al. 2018). Severe forest disturbances such as clearcutting and understory removal can therefore be expected to reduce fungal richness and alter community composition both in tropical and temperate regions (Tedersoo et al. 2014; Zhao et al. 2011), with generalist and opportunistic fungi often proliferating following vegetation loss (Mueller et al. 2016).

Arbuscular mycorrhizal fungi (AMF) (phylum Glomeromycota) play a particularly important role in terrestrial ecosystems, as they form mutualistic associations with about 80% of all terrestrial plants (Smith and Read 2008; Moora et al. 2014; Brundrett and Tedersoo 2018). They facilitate plant nutrient uptake, especially phosphorus, in exchange for photosynthetically derived carbon, thereby enhancing plant growth (Dai et al. 2023). They also improve tolerance to biotic and abiotic stresses, including drought, salinity, and pathogens (Gianinazzi et al. 2010; Jung et al. 2012). AMF also contribute to soil carbon sequestration (Frey 2019) and the maintenance of soil structure (Rillig and Mummey 2006; Bedini et al. 2009), thus promoting climate regulation. Because AMF depend on host plants, alterations in vegetation composition and soil disturbance can be expected to disrupt their abundance and community structure (Thormann et al. 1999; Sepp et al. 2018). However, the response to disturbances has been shown to vary among AMF lineages: taxa with high growth rates and short life cycles from the family Glomeraceae often dominate agricultural soils (Jansa et al. 2002; Chagnon et al. 2013; Thanni et al. 2022), while taxa from the families Gigasporaceae and Acaulosporaceae are more sensitive to soil disturbance (Ohsowski et al. 2014; Van Der Heyde et al. 2017).

Despite the ecological significance of AMF and fungi in general, studies on how land‐use change affects fungal communities in African tropical forest soils remain limited (Guidosse et al. 2025; Olanipon et al. 2024). Our previous study in a single forest ecosystem (Ise Forest Reserve, southwestern Nigeria) used high‐throughput sequencing to characterize AMF communities and revealed high AMF diversity forming dense networks with tropical tree species (Olanipon et al. 2024). However, that study focused exclusively on AMF but did not examine total fungal communities, functional guilds (saprotrophs, pathogens), or compare different land‐use types. Other studies in West Africa have addressed specific aspects of fungal ecology, but important knowledge gaps remain. For example, Kumah et al. (2025) used high‐throughput sequencing to characterize fungal diversity in agroforestry farms in Ghana and Côte d'Ivoire, but restricted their analysis to cocoa systems. Meidl et al. (2021) characterized ectomycorrhizal fungal communities across five West African countries (Benin, Burkina Faso, Mali, Guinea, and Côte d'Ivoire) but limited their study to woodland ecosystems. Olou et al. (2019) investigated wood‐inhabiting fungi across a climatic gradient in Benin, but did not examine soil fungi or land‐use effects while Tchabi et al. (2008) assessed AMF communities in sub‐Saharan savannas of Benin under different agricultural land‐use intensities, but relied on spore‐based morphological identification rather than high‐throughput sequencing.

In this study, we used high‐throughput amplicon sequencing to characterize total soil fungal communities, including AMF, saprotrophs, and pathogens, across three land‐use types (undisturbed forest, disturbed forest, and croplands) in two forest reserves (Akure and Oba Hills) in southwestern Nigeria. Forests in this region are increasingly fragmented and degraded due to agricultural expansion, urbanization, and timber harvesting (Nwoboshi 1989; Nwabueze et al. 2023), yet the consequences of these disturbances for belowground fungal communities and associated ecosystem functions remain poorly understood. Specifically, we aimed to (i) characterize fungal taxa associated with soils collected from both forest reserves and (ii) assess the effects of land‐use change on soil fungal diversity, community composition, and key functional groups. We hypothesized that cropland and disturbed forest soils would host less diverse and more homogeneous fungal assemblages than undisturbed forest soils; that AMF and saprotrophic fungal richness would be higher in undisturbed forest soils due to greater plant diversity and litter inputs; and that conversion to cropland would favor more ruderal, disturbance‐tolerant AMF taxa, increase plant pathogen diversity, and alter saprotrophic fungal communities.

## 2 Materials and Methods

### 2.1 Study Sites

**FIGURE 1:**
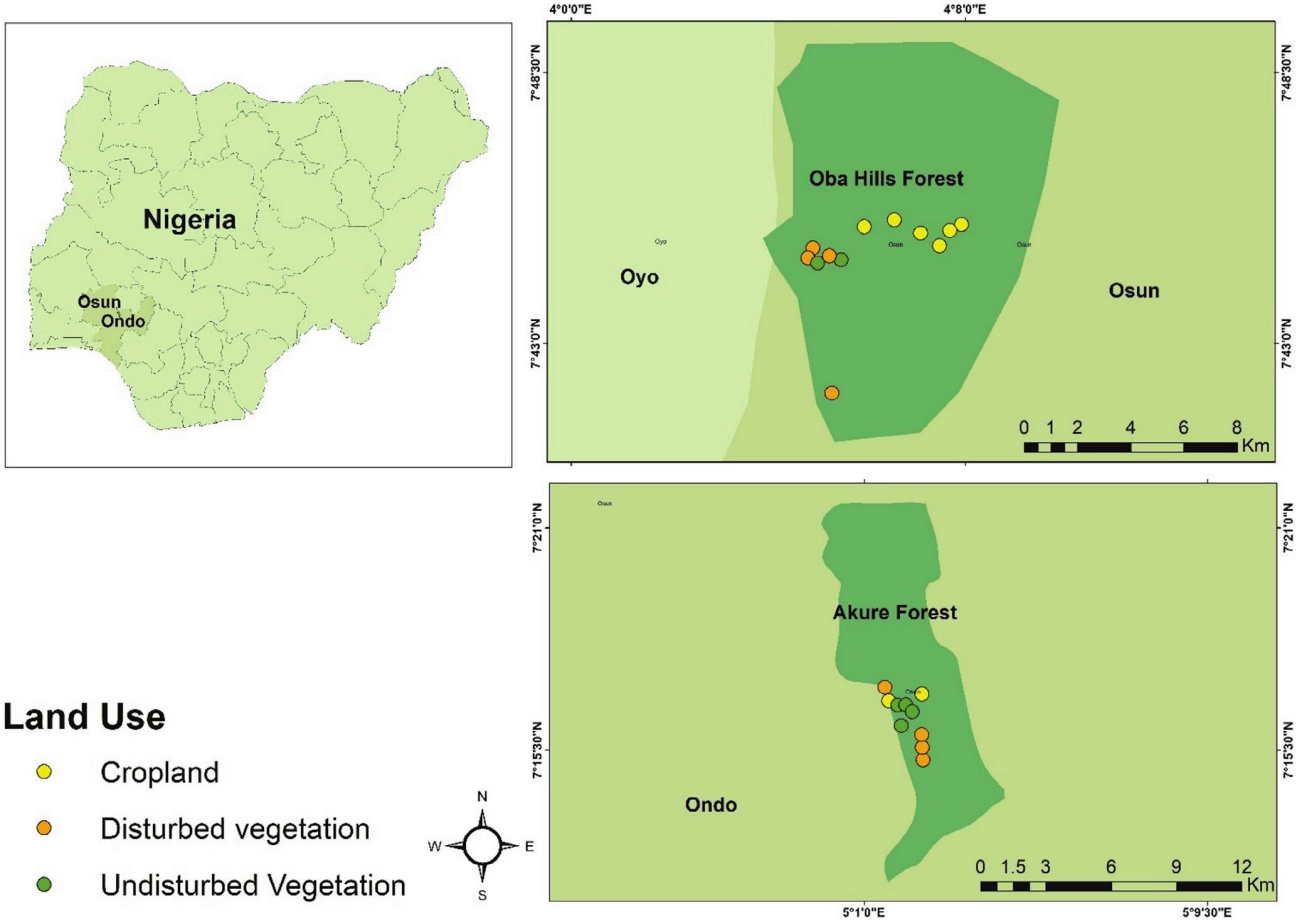
Map showing the location of the Akure Forest Reserve (Ondo State) and Oba Hills Forest Reserve (Osun State) in southwestern Nigeria. Sampling sites across different land‐use types (undisturbed forest, plantations, and croplands) are indicated.

The study was conducted in two tropical rainforest reserves in Southwest Nigeria: Akure Forest Reserve (Ondo State) and Oba Hills Forest Reserve (Osun State) (Figure 1). Both reserves lie within the tropical humid climatic zone, characterized by distinct wet and dry seasons, and support diverse vegetation types ranging from undisturbed forest stands to plantations and croplands. Each reserve also contains protected areas with high floristic and faunal diversity (Adekunle et al. 2013; Wahab et al. 2021). Despite ongoing protection efforts, both reserves have been subjected to significant anthropogenic activities, particularly agriculture, logging, and deforestation, which have led to fragmentation and degradation of forest habitats.

**FIGURE 2:**
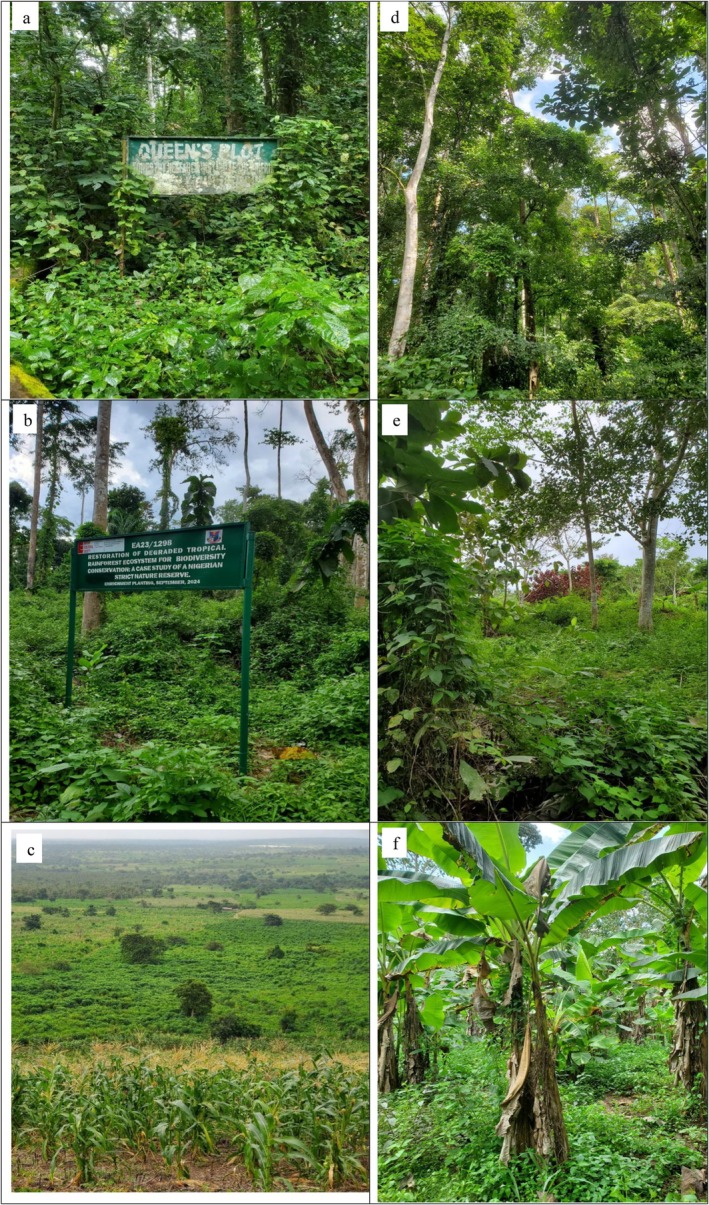
Representative photographs of the different land‐use types sampled in the Akure and Oba Hills Forest Reserves: (a, d) undisturbed forest, (b, e) disturbed forest (plantations), and (c, f) croplands.

Akure Forest Reserve (7°16.679′ N, 5°2.216′ E, 253 m a.s.l) covers an area of approximately 69.93 km^2^ (Oguntimehin and Onyekwelu 2019) and experiences an average annual rainfall of about 1700 mm and a mean temperature of 27.1°C (Akinbowale et al. 2020). The undisturbed section of the forest, known as the Queen's Plot, consists mainly of broad‐leaved hardwood species forming a dense canopy (Adekunle et al. 2013) (Figure 2a). Dominant tree species include *Brachystegia nigerica*, *Chrysophyllum albidium*, *Mansonia altissima*, *Blighia sapida* , *Ficus mucuso*, *Triplochiton scleroxylon*, *Ricinodendron heudelotii, Funtumia elastica*, *Terminalia superba* , *Ceiba pentandra* , *Celtis zenkeri*, *Pterygota macrocarpa*, *Albizia zygia* , and *Dacryodes edulis* . Disturbed areas contain plantations of *Tectona grandis* and *Gmelina arborea* , together with sparse native trees and herbaceous species including *Sida acuta* , *Talinium triangulare*, *Chromolaena odorata* , and *Tithonia diversifolia* (Figure 2b). Croplands include mixed farming systems with *Colocasia esculenta* (cocoyam), Musa spp. (banana and plantain), *Oryza sativa* (rice), *Theobroma cacao* (cocoa), and *Zea mays* (maize) (Figure 2c).

Oba Hill Forest reserve (7°41′25′N—7°49′15′N; 4°4′10′E—4°7′30′E), which is a part of the Oba Hills National Park covers about 49.3 km^2^ and includes three prominent hills rising up to 401 m a.s.l., interspersed with ridges and valleys. The mean annual rainfall is approximately 1350 mm, and the average temperature is 27°C (Akinsanola and Ogunjobi 2014; Adeyemi and Ayinde 2022). Undisturbed areas are dominated by tree species such as *Senna siamea* , *Azadirachta indica* , *Funtumia elastica, Terminalia glaucescens*, *Ceiba pentandra* , *Cola gigantea*, *Bosqueia angolensis*, *Cleistopholis patens*, *Newbouldia laevis*, and *Spondias mombin* (Figure 2d). Disturbed areas consist mainly of plantations of *Gmelina arborea* , *Elaeis guineensis* , and *Tectona grandis* (Figure 2e) with sparse native trees and understory vegetation. Other parts of the reserve are used as croplands planted with *Zea mays (*maize), *Vigna unguiculata* (beans), *Sorghum bicolor* (pearl millet), and *Manihot esculenta* (cassava), as well as extensive grasslands dominated by *Pennisetum purpureum* and *Eleusine indica* . Common herbaceous species in plantations and farming systems include *Ageratum conyzoides* , *Sida acuta* , *Chromolaeena odorata*, *Acmella oleracea*, *Tithonia diversifolia* , *Calotropis procera* , *Vernonia amygdalina*, and *Euphorbia hirta* (Figure 2f).

### 2.2 Sample Collection

Soil sampling was conducted between the last week of October and the first week of November 2024, following the protocol of the Society for the Protection of Underground Networks (SPUN). Within each forest reserve, land‐use types were classified based on the level of anthropogenic disturbance: plots retaining their original vegetation were classified as undisturbed forest, those with clear evidence of deforestation, afforestation with tree plantations, or soil tillage were classified as disturbed forest, and areas showing evidence of degradation due to farming activities were classified as croplands.

In Akure Forest Reserve, a total of 12 undisturbed forest, 7 disturbed forest, and 11 cropland plots were sampled. In Oba Hills Forest Reserve, 11 undisturbed forest, 13 disturbed forest, and 11 cropland plots were sampled. Following the SPUN sampling protocol, at each plot, two transects were laid at right angles to delineate a 30 m × 30 m plot (Figure S1). Before sampling, leaf litter was carefully removed using a shovel. Within each plot, nine soil sub‐samples (Figure S1) were collected with a 5 cm diameter metal soil corer at a depth of 0–10 cm, as this layer harbors the majority of the fungal communities of interest in this study (Manral et al. 2022). This depth was selected because it represents the zone of highest root density, organic matter accumulation, and microbial activity, where microbial biomass is typically greatest in nutrient‐rich surface layers of forest soils (Frymark‐Szymkowiak and Karliński 2022). It also harbors the highest fungal biomass and diversity, which generally decrease with increasing soil depth (Mundra et al. 2021).

The nine sub‐samples collected from each plot were thoroughly mixed to form a composite sample. Visible litter and roots were manually removed, and the soil was passed through a 2 mm mesh sieve to obtain a homogenized sample, which was stored in Ziploc bags. Because of the extended sampling period, the remoteness of the study sites, and limited access to electricity, samples were air‐dried at room temperature for 48 h prior to DNA extraction. Although air‐drying is not the preferred method for preserving soil microbial DNA, recent work has reported no significant differences in fungal diversity or community composition between air‐dried and frozen soil samples (Edwards et al. 2024; Han et al. 2026). Hence, air‐drying soil samples is the best available method under the local circumstances.

### 2.3 Soil Properties Analyses

Soil samples were analyzed for pH, available phosphorus, total nitrogen, exchangeable bases (K, Ca, Mg), cation exchange capacity (CEC), total organic carbon (TOC), and soil texture using standard laboratory methods. Soil pH was measured using a glass electrode in a 1:2.5 soil‐to‐water suspension (Peech 1965). Available phosphorus was extracted using 2 M HCl and determined colorimetrically using the vanadate‐molybdate method at 470 nm (Bray and Kurtz 1945). Total nitrogen (TN) was determined using standard digestion procedures, while potassium and exchangeable bases were measured using a Jenway PFP7 digital flame photometer (Fisher Scientific, USA). Total organic carbon was determined using the Walkley‐Black wet‐oxidation method, in which organic carbon is oxidized with potassium dichromate and sulfuric acid followed by titration (Walkley and Black 1934). CEC was determined using the ammonium acetate method, and the carbon‐to‐nitrogen (C:N) ratio was calculated as the ratio of total organic carbon to total nitrogen. Soil texture was determined using the hydrometer method (Bouyoucos 1962).

### 2.4 DNA Extraction, PCR Amplification, and High‐Throughput Sequencing

Total genomic DNA was extracted in duplicate from 250 mg of air‐dried soil using the GeneMATRIX Soil DNA Purification Kit (EurX, Gdańsk, Poland), following the manufacturer's instruction. DNA concentration and purity were assessed using a Nanodrop One spectrophotometer (Thermo Fisher Scientific Inc., USA). Duplicate DNA extracts from each sample were pooled and stored at −80°C until further processing.

The fungal ITS2 region was amplified using primers ITS3 (5′–GCATCGATGAAGAACGCAGC–3′) and ITS4 (5′–TCCTCCGCTTATTGATATGC–3′) under the following PCR conditions: initial denaturation at 95°C for 2 min, followed by 40 cycles of denaturation at 95°C for 30 s, annealing at 55°C for 30 s, and extension at 72°C for 1 min, followed by a final extension phase at 72°C for 10 min (Op De Beeck et al. 2014).

To specifically target arbuscular mycorrhizal fungi (Glomeromycota), the 18S SSU rDNA region was amplified using the primer pair WANDA (5′–CAG CCG CGG TAA TTC CAG CT–3′) and AML2 (5′–GAACCCAAACACTTTGGTTTCC–3′) under the following PCR conditions: initial denaturation at 95°C for 2 min; 30 cycles of denaturation at 95°C for 30 s, annealing at 54°C for 40 s and extension at 72°C for 1 min, followed by a final extension at 72°C for 10 min (Kajihara et al. 2022).

The ITS2 and SSU libraries were pooled, purified using a 1× AMPure XP bead cleanup, eluted in 30 μL of nuclease‐free water, and verified on a 2% agarose gel. The quality of the amplicon libraries was further assessed using an Agilent Bioanalyzer 2100. Sequencing of the ITS2 and SSU amplicons was performed on an Illumina NextSeq 2000 platform 2 × 150 bp at the Quadram Institute Bioscience (QIB), Norfolk, UK.

For total fungal community analysis, we used the ITS2 marker because of its broad taxonomic coverage and suitability for identifying major fungal taxa and functional guilds, including saprotrophs and plant pathogens. However, as this marker has been shown to be suboptimal to characterize AMF (Stockinger et al. 2010), we used the SSU (18S rRNA) marker targeting Glomeromycota. The SSU region produces robust resolution for AMF but captures non‐AMF diversity less effectively. Consequently, ITS and SSU sequence data were analyzed separately, and direct comparisons of richness between AMF ASVs and fungal OTUs were avoided because of differences in primer specificity, amplicon length, and taxonomic coverage.

### 2.5 Bioinformatic Analyses

High‐throughput sequencing data from the ITS2 and SSU regions were processed using the LotuS2 pipeline (Özkurt et al. 2022). For ITS2 data, DADA2 (Callahan et al. 2016) was used for error correction and ASV inference, after which ASVs were clustered into Operational Taxonomic Units (OTUs) at 97% sequence similarity using VSEARCH (Rognes et al. 2016). For SSU data, DADA2‐derived ASVs were retained without clustering because ASVs provide higher taxonomic resolution for AMF than OTU‐based approaches (Callahan et al. 2017; Edgar 2018). Raw paired‐end reads were demultiplexed based on sample‐specific barcodes and primer sequences. Reads were then merged, quality‐filtered, and clustered into Operational Taxonomic Units (OTUs) and Amplicon Sequence Variants (ASVs) for ITS2 (total fungal community) and SSU (arbuscular mycorrhizal fungi) datasets, to reduce noise and mitigate sequencing errors. Potential contaminant sequences were identified and removed using the *Decontam R* package based on negative control samples. To minimize potential artifacts introduced during PCR and sequencing, OTUs representing < 0.01% of the total reads per sample were further filtered out (Alberdi et al. 2018). Taxonomic assignment for ITS2 OTUs was performed against the UNITE fungal ITS reference database (Kõljalg et al. 2013). SSU sequences were assigned using the MaarjAM database of published Glomeromycota sequences (Öpik et al. 2010). Raw sequencing data was submitted to the NCBI Sequence Read Archive and is available under Bioproject PRJNA1396320. OTUs from the fungal ITS2 dataset were classified into ecological groups based on their genus‐level identifications using the FungalTraits database (Põlme et al. 2020). Two subsets of the ITS2 dataset were created based on these ecological classifications: one containing OTUs that had “plant_pathogen” as primary lifestyle according to FungalTraits and one containing OTUs that had “soil_saprotroph”, “litter_saprotroph”, “wood_saprotroph”, or “unspecified_saprotroph” as primary lifestyle (hereafter referred to as the saprotrophic fungi).

### 2.6 Statistical Analysis

All statistical analyses and data visualization were performed in R Studio (R Core Team 2023; R version 4.3.2). Rarefaction curves were generated for the complete ITS2 and SSU datasets using the “*rarefy*” function to ensure that sequencing depth was sufficient to capture the fungal diversity of each sample. Bar plots representing the relative abundance of sequence reads for the total fungal community (class level), AMF community (genus level), plant pathogenic fungi (genus level) and saprotrophic fungi (genus level) were created using the “*ggplot2*” package (Wickham 2016).

A linear model was used to compare OTU and ASV richness of the total fungal communities, AMF communities, plant pathogenic fungal communities and saprotrophic fungal communities between the three land use types (cropland, disturbed forest and undisturbed forest) and the two study sites (Akure Forest Reserve and Oba Hills Forest Reserve). Statistical significance of the variables was tested using type II tests with the *car* package (Fox and Weisberg 2019). The effect of land‐use on the total fungal, AMF, plant pathogenic fungal and saprotrophic fungal community composition was tested using permutational analysis of variance (PERMANOVA) with the “*adonis2*” function in the “*vegan*” package in R (Oksanen et al. 2004). Non‐metric multidimensional scaling (NMDS), based on the Bray‐Curtis distances, was used to visualize differences in total fungal, AMF, plant pathogenic fungal and saprotrophic fungal community composition. NMDS stress values were calculated to assess ordination reliability, where stress < 0.2 indicates an acceptable fit. In cases where the stress values were too high, a principal coordinates analysis (PCoA) was used instead. Redundancy analysis (RDA) on Hellinger‐transformed ASV/OTU tables was used to elucidate the relationships between soil variables and fungal community composition (Martiny et al. 2011). Finally, indicator species analysis was performed on the ITS dataset using the “*indicspecies*” package (De Cáceres et al. 2016) to identify OTUs specifically associated with each land‐use type.

## 3 Results

### 3.1 Soil Physicochemical Properties

Soil physicochemical properties varied across land‐use types and between the two forest reserves (Table 1). Soil pH was slightly acidic to near neutral across all land use types (pH 5.9–6.5). Total nitrogen, available phosphorus, potassium, calcium, magnesium, cation exchange capacity, and total organic carbon were consistently higher in undisturbed forests compared to disturbed forests and croplands. The carbon‐to‐nitrogen ratio was highest in undisturbed forest soils in Akure (14.66) and croplands in Oba Hills (9.78). Soil texture also differed between the two forest reserves. Soils in Akure Forest Reserve were characterized by higher sand content (50.45%–58.62%) compared to Oba Hills Forest Reserve (45.96%–52.81%). In Akure Forest Reserve, undisturbed forest soils had the highest silt content (35.01%) and lowest clay content (14.55%), whereas in Oba Hills Forest Reserve, croplands had the lowest sand content (45.96%) and disturbed forest soils had the highest clay content (19.39%).

**TABLE 1:** Soil physicochemical properties and plant diversity indices under different land‐use types in the study sites.

	Akure forest reserve			Oba hills forest reserve		
	Cropland	Disturbed forest	Undisturbed forest	Cropland	Disturbed forest	Undisturbed forest
pH	6.21 ± 0.04	6.09 ± 0.15	6.33 ± 0.03	6.08 ± 0.08	5.91 ± 0.09	6.50 ± 0.82
TN (%)	0.15 ± 0.01	0.13 ± 0.01	0.23 ± 0.01	0.24 ± 0.0	0.25 ± 0.02	0.39 ± 0.01
P (mg/kg)	15.93 ± 0.15	15.52 ± 1.74	22.91 ± 1.11	21.74 ± 0.64	24.69 ± 0.71	29.45 ± 0.49
K (mg/kg)	24.4 ± 2.4	28.03 ± 1.52	45.42 ± 1.72	31.8 ± 1.53	40.6 ± 2.29	53.6 ± 1.16
Ca (mg/kg)	69.13 ± 2.01	54.53 ± 3.39	81.45 ± 3.0	118.13 ± 1.43	139.53 ± 7.46	167.03 ± 3.77
Mg (mg/kg)	17.09 ± 1.34	20.76 ± 1.22	27.13 ± 0.73	24.59 ± 0.45	25.25 ± 2.15	34.46 ± 1.3
CEC (cmolc/kg)	11.74 ± 0.7	12.04 ± 1.06	15.48 ± 0.51	16.5 ± 0.77	16.43 ± 1.24	22.94 ± 0.75
TOC (%)	1.92 ± 0.04	1.92 ± 0.14	2.51 ± 0.01	2.38 ± 0.05	2.17 ± 0.07	3.73 ± 0.08
C:N	13 ± 0.1	14.66 ± 1.69	10.83 ± 0.5	9.78 ± 0.08	9.13 ± 1.1	10.78 ± 0.68
Sand (%)	53.18 ± 1.68	58.62 ± 1.16	50.45 ± 1.14	45.96 ± 1.25	52.81 ± 3.32	49.31 ± 1.19
Silt (%)	21.32 ± 3.13	20.47 ± 2.19	35.01 ± 1.28	35.54 ± 2.95	27.81 ± 0.53	35.2 ± 3.55
Clay (%)	24.87 ± 1.45	20.92 ± 1.03	14.55 ± 2.42	16.99 ± 2.69	19.39 ± 2.8	15.5 ± 2.36
Species richness	6.4 ± 1.03	4.46 ± 0.68	9.25 ± 0.68	5.05 ± 0.44	5.2 ± 1.02	3.82 ± 0.23
Shannon index	1.81 ± 0.16	1.32 ± 0.18	2.2 ± 0.07	1.54 ± 0.1	1.57 ± 0.21	1.32 ± 0.06

*Note:* Plant diversity indices represent species richness and Shannon diversity index per 30 m × 30 m plot under the different land use types. Values are means ± standard error.

Abbreviations: C:N, carbon‐to‐nitrogen ratio; Ca, calcium; CEC, cation exchange capacity; K, potassium; Mg, magnesium; P, available phosphorus; TN, total nitrogen; TOC, total organic carbon.

### 3.2 Taxonomic Diversity and Composition

After quality filtering and removal of chimeras, a total of 18,335,945 ITS2 and 13,788,032 SSU high‐quality sequences were retained for downstream analyses. Rarefaction curves indicated that sequencing depth was sufficient to capture the fungal diversity in all soil samples (Figures S2 and S3).

**FIGURE 3:**
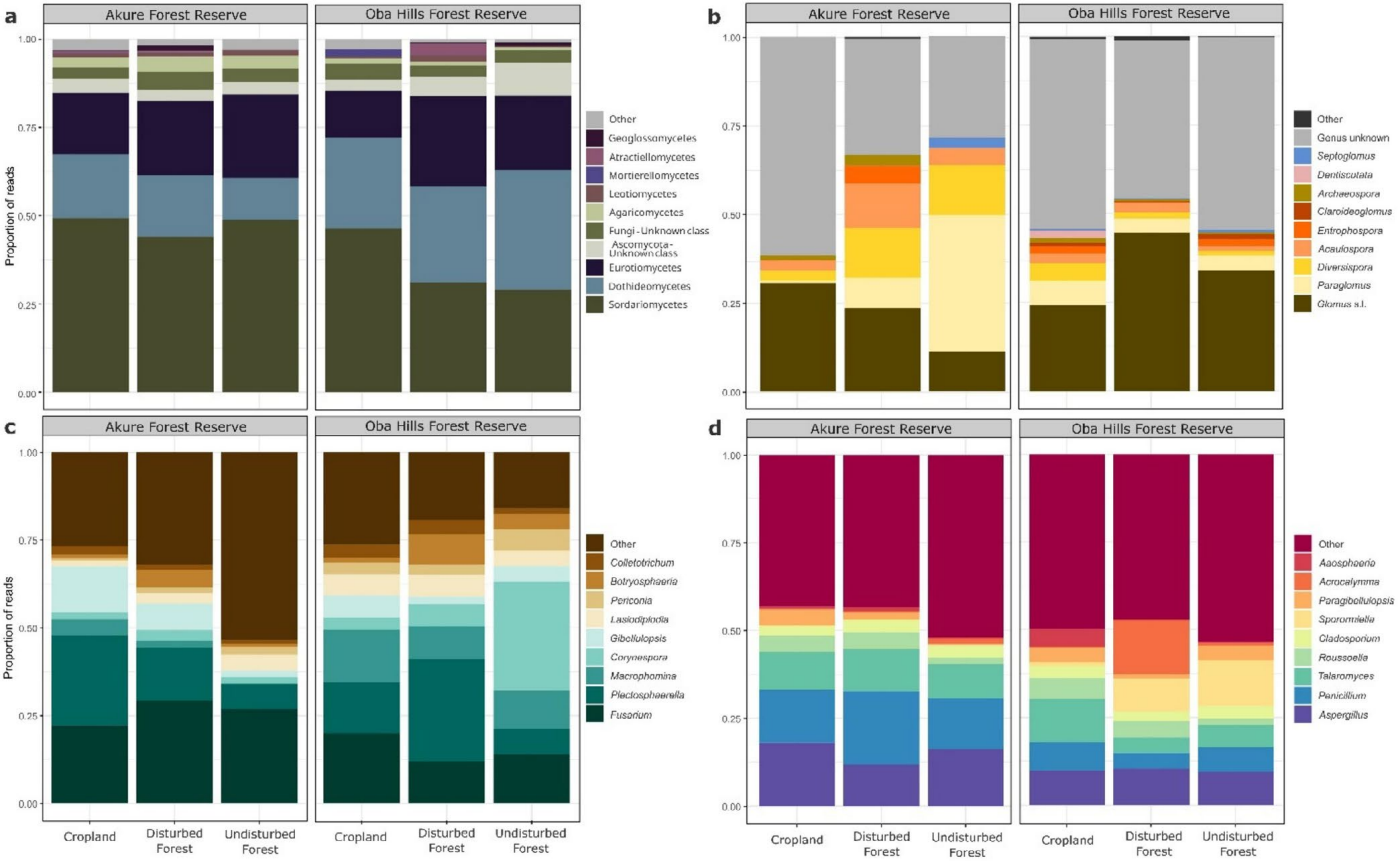
Taxonomic composition of fungal communities across study sites and land‐use types. (a) Relative abundance of fungal classes in total fungal communities (ITS2 dataset) from soil samples collected in Akure Forest Reserve and Oba Hills Forest Reserve under cropland, disturbed forest, and undisturbed forest land uses. (b) Relative abundance of arbuscular mycorrhizal fungal (AMF) genera (SSU dataset) in the same samples. Unknown or unclassified taxa are indicated as “Unknown class” or “Genus unknown,” respectively. (c) composition of the plant pathogenic genera (a subset of the ITS2 dataset). (d) composition of the saprotrophic fungal genera (a subset of the ITS2 dataset).

Across the soil samples, a total of 9280 fungal OTUs (ITS2) and 738 AMF ASVs (SSU) were obtained. Taxonomic profiling of the total fungal community revealed that sequences were dominated by the classes Sordariomycetes, Eurotiomycetes, and Dothideomycetes, with only a small fraction remaining unclassified (Figure 3a). Among AMF, *Glomus* s.l. was the predominant genus across all samples, followed by *Paraglomus*, *Diversispora*, and *Acaulospora*. Distinct genera such as *Claroideoglomus* and *Dentiscutata* were observed predominantly in Oba Hills Forest Reserve samples (Figure 3b). The most abundant plant pathogenic fungal genera were *Fusarium*, *Plectosphaerella* and *Macrophomina* (Figure 3c), while the most abundant saprotrophic genera were *Aspergillus*, *Penicillium*, and *Talaromyces* (Figure 3d).

### 3.3 Total Fungal Diversity and Community Composition

**FIGURE 4:**
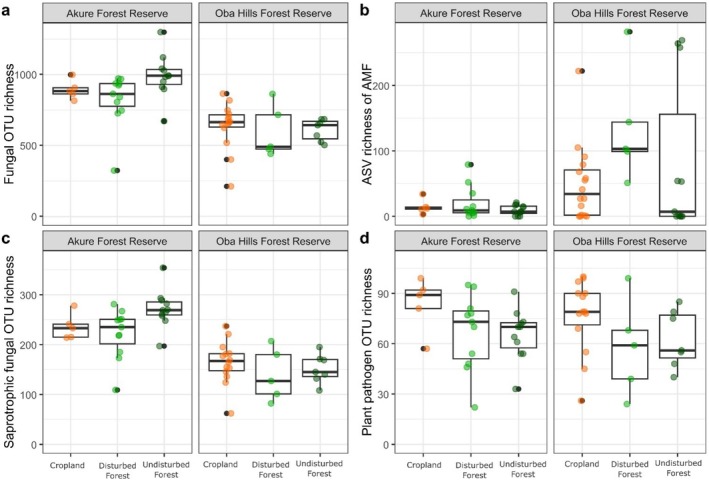
Operational Taxonomic Unit (OTU) richness of total fungal communities (a), of saprotrophic fungal communities (c) and of plant pathogenic fungi (d) and AMF Amplicon Sequence Variant (ASV) richness (b) across land‐use types in Akure Forest Reserve and Oba Hills Forest Reserve. Boxplots show the variation in fungal OTU richness among cropland, disturbed forest, and undisturbed forest sites. Each dot represents an individual soil sample. Richness was significantly different between the two forest reserves but did not differ significantly across land‐use types (see Section 3 for statistical details).

The linear model showed that fungal OTU richness was significantly different between the two study sites (*F*_1,49_ = 41.0, *p* < 0.001), with higher richness observed in Akure Forest Reserve compared to Oba Hills Forest Reserve. In contrast, land‐use type had no significant effect (*F*_2,49_ = 2.3, *p* = 0.116), although there was a trend towards higher fungal OTU richness in undisturbed forests and lower in disturbed forests across both sites (Figure 4a).

For AMF, ASV richness was significantly affected by study site (*F*_1,56_ = 16.2, *p* < 0.001) but not by land‐use type (*F*_2,56_ = 2.1, *p* = 0.129). Akure Forest Reserve consistently showed lower AMF richness across all land‐use types compared to Oba Hills Forest Reserve (Figure 4b). Saprotrophic and plant pathogenic fungal OTU richness both differed significantly among land‐use types (*F*_2,49_ = 3.2, *p* = 0.047 and *F*_2,49_ = 3.6, *p* = 0.034, respectively). Saprotrophic fungal richness was highest in undisturbed forests and lowest in disturbed forests; croplands did not differ from either forest type. Plant pathogenic fungal richness was highest in croplands (Figure 4c,d). Saprotrophic fungal richness was also significantly higher in the Akure Forest Reserve than in the Oba Hills Forest Reserve (*F*_1,49_ = 55.9, *p* < 0.001), whereas no significant difference between sites was observed for plant pathogenic fungi (*F*_1,49_ = 1.1, *p* = 0.304).

### 3.4 Fungal Diversity and Community Composition

**FIGURE 5:**
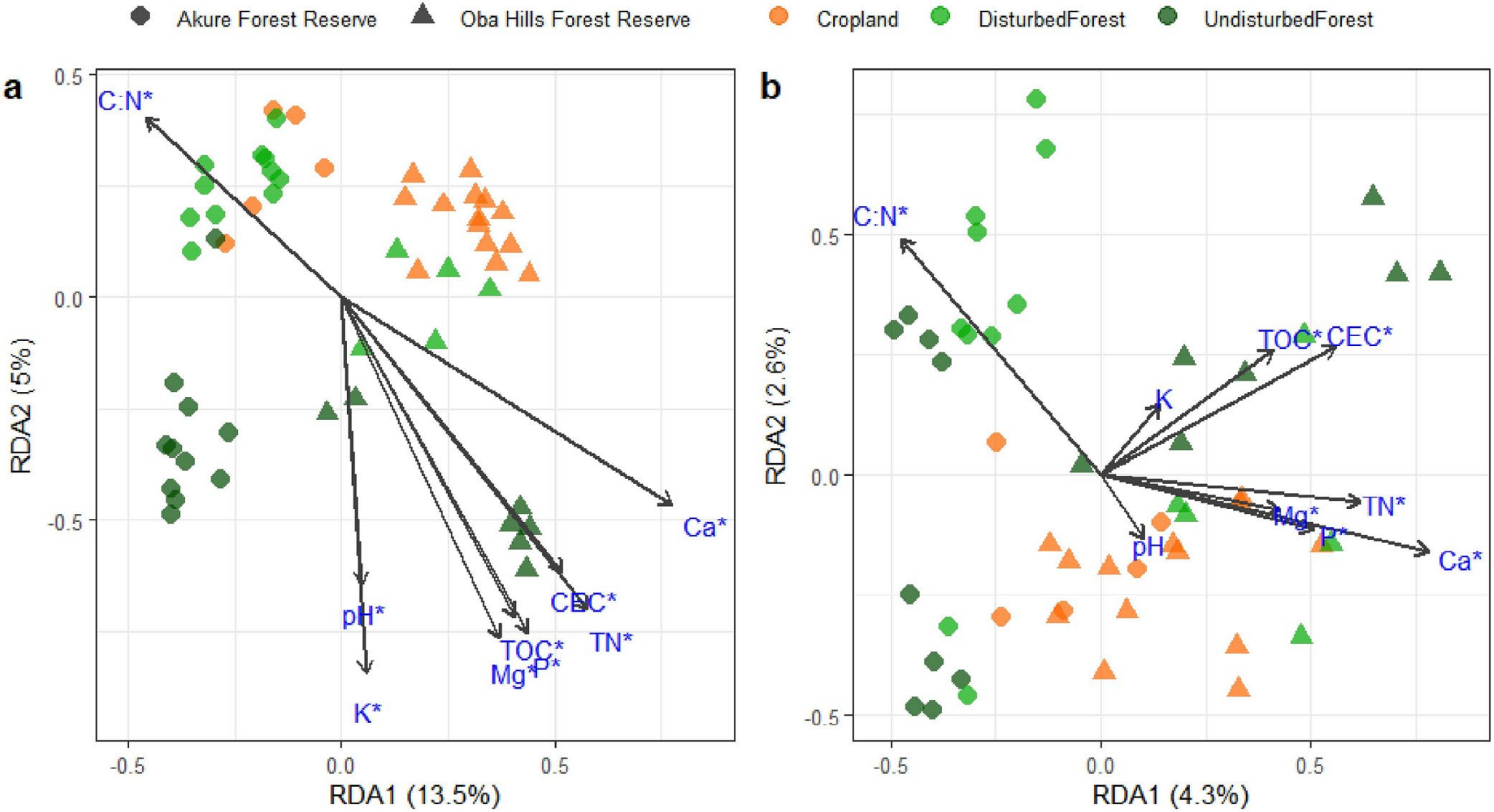
Redundancy analyses of soil fungal community composition (a) and of arbuscular mycorrhizal fungal communities (b) across land use types in the two study sites, with soil variables overlaid as explanatory vectors. RDA1 and RDA2 represent proportions of variation in fungal community explained by constrained axes. Arrows indicate the direction and strength of soil variables. Both total fungal and arbuscular mycorrhizal fungal communities were significantly influenced by all measured soil variables (*p* < 0.05). For unconstrained ordinations of all soil fungi, AMF and the saprotrophic and plant pathogenic fungi, see Figure S4.

A PERMANOVA revealed that both land‐use type (*F* = 2.01, *R* ^2^ = 0.066, df = 2, *p* = 0.032) and study site (*F* = 6.87, *R* ^2^ = 0.112, df = 1, *p* < 0.001) had a significant effect on total fungal community composition. NMDS ordination based on the Bray‐Curtis distances showed clear clustering of fungal communities by study site (stress = 0.116 for total fungi), with partial overlap between the disturbed and undisturbed forests, and between the disturbed forest and cropland (Figure 5a). Similarly, plant pathogenic and saprotrophic fungal communities significantly differed among land‐use types (*F* = 3.1, *R* ^2^ = 0.089, *p* < 0.001 and *F* = 2.6, *R* ^2^ = 0.085, *p* < 0.001, respectively) and study sites (*F* = 10.5, *R* ^2^ = 0.153, *p* < 0.001 and *F* = 6.1, *R* ^2^ = 0.099, *p* < 0.001, respectively), resulting in similarly looking NMDS ordinations (Figure S4). For AMF, community composition significantly differed between study sites (*F* = 2.1, *R* ^2^ = 0.119, df = 1, *p* < 0.001) but not by land‐use type (*F* = 1.1, *R* ^2^ = 0.039, df = 2, *p* = 0.351). As the stress value of an NMDS on AMF was considered too high (0.248), a PCoA was plotted instead. The PCoA ordination indicated some separation between samples from Akure and Oba Hills Forest Reserves, with overlap among the land‐use types (Figure S4).

To further understand the relationship between fungal community composition and soil variables, a redundancy analysis was performed (Figure 5). The results indicated that the measured soil variables significantly influenced both total fungal (pseudo‐*F* = 2.23, *p* = 0.001) and arbuscular mycorrhizal fungal community compositions (pseudo‐*F* = 1.18, *p* = 0.001). For the total fungal community, the first and second RDA axes represented 13.5% and 5% of the constrained variation, respectively (Figure 5a). Fungal communities in croplands were mostly positively associated with Ca, CEC, TN, TOC, Mg, and available P, whereas undisturbed forests were associated with higher C:N ratios. Soil pH also significantly influenced the total fungal communities, although its effect was weaker in comparison to soil fertility indicators. On the other hand, the first two axes explained only 6.9% of variation in AMF community composition (RDA1 = 4.3%; RDA2 = 2.6%) (Figure 5b). Although Ca, CEC, TOC, TN, P, and Mg remained positively associated, there was considerable overlap among land‐use types. Soil pH also had a significant but weaker effect on AMF community composition.

### 3.5 Indicator Species Analysis

Indicator species analysis identified significant associations between fungal OTUs and land‐use types. Of the 9280 fungal OTUs detected, 170 were significantly associated with cropland, 171 with disturbed forest, and 333 with undisturbed forest. The number of indicator taxa increased with decreasing land‐use intensity, with the highest number of indicator taxa associated with undisturbed forests, followed by disturbed forests and croplands. Additionally, 80 OTUs were shared between cropland and disturbed forest, 113 between disturbed and undisturbed forest, and 12 between cropland and undisturbed forest ecosystems.

**FIGURE 6:**
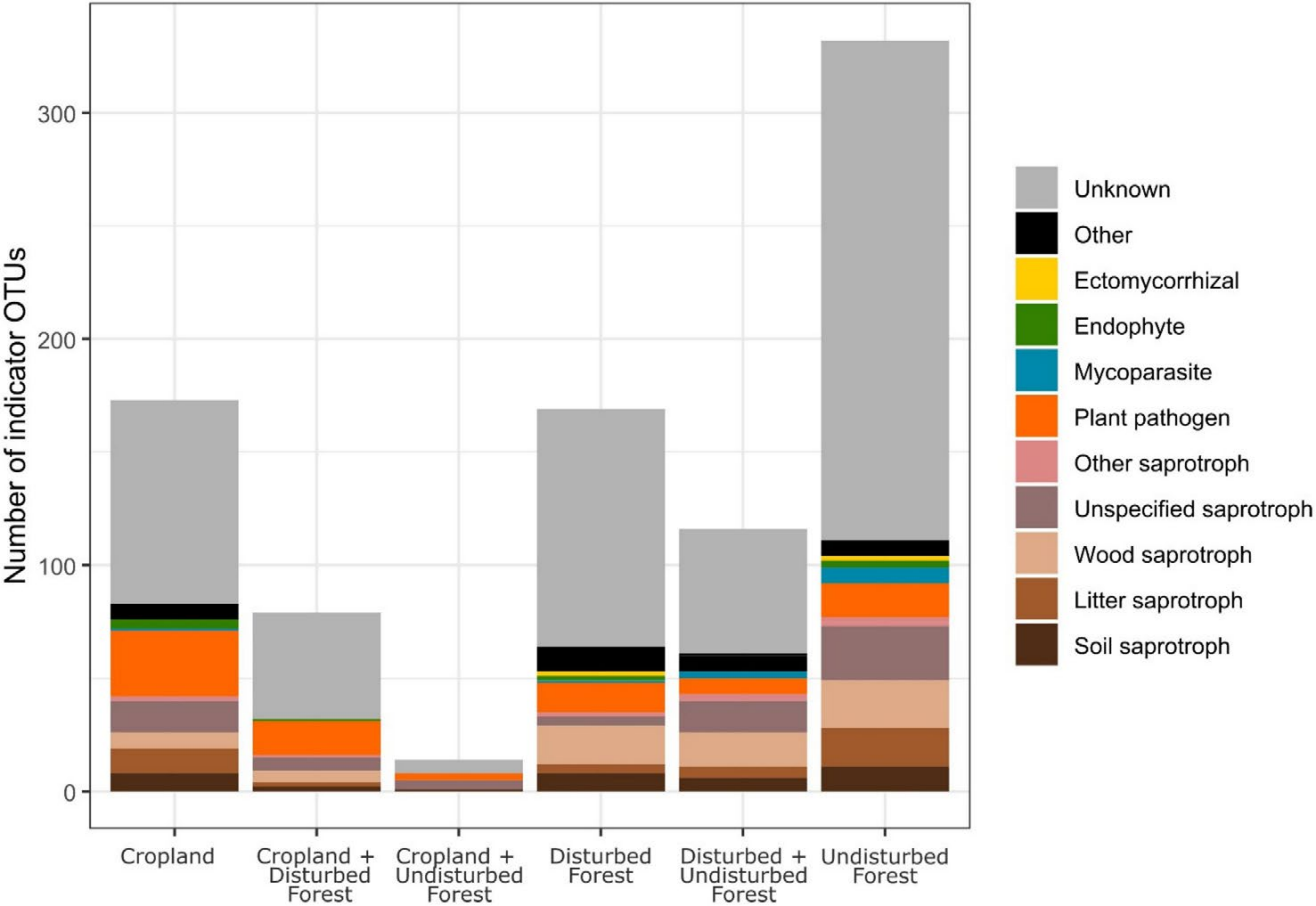
Indicator OTUs assigned to each of the land use types and their combinations, colored according to their ecology. 170 OTUs were indicative for cropland, 80 OTUs for cropland and disturbed forest, 12 OTUs for cropland and undisturbed forest, 113 OTUs for disturbed and undisturbed forest, 171 OTUs for disturbed forest, and 333 for undisturbed forest.

Although most indicator OTUs could not be assigned to an ecological guild, those that were classified showed clear functional differences among land use types. Indicator OTUs associated with croplands were predominantly plant pathogens, whereas undisturbed forests were mainly characterized by wood‐ or litter‐decaying fungi. Disturbed forests showed a more heterogenous functional composition, including saprotrophs, pathogens, and mycoparasites (Figure 6). OTUs shared between cropland and disturbed forests were primarily pathogens, while those shared between disturbed and undisturbed were mainly saprotrophs.

## 4 Discussion

Land‐use change, particularly the conversion of natural forests into agricultural landscapes, is a major driver of biodiversity loss and soil degradation globally (Holden and Treseder 2013; Mueller et al. 2016). West African tropical forests, including those in Nigeria, are experiencing rapid fragmentation and degradation, primarily driven by human activities such as logging, agricultural expansion, and bush burning (Nwabueze et al. 2023; Nwoboshi 1989; Olanipon et al. 2025; Ologunde et al. 2025). Given the critical role of soil microbial communities in maintaining ecosystem functioning, understanding how fungal and arbuscular mycorrhizal (AMF) communities respond to these anthropogenic factors is essential for integrating tropical forest conservation with sustainable land management.

### 4.1 Fungal Diversity

Consistent with studies from other tropical systems (Adeboye et al. 2011; Klironomos et al. 2011; Adekunle et al. 2013; Brinkmann et al. 2019; Medriano et al. 2023), we found high fungal and AMF diversity in both forest reserves. Fungal diversity is generally higher in tropical than in temperate forests, likely due to the combined effects of high plant diversity, stable climatic conditions, and favorable soil physicochemical properties that support fungal growth.

High plant species richness in tropical forests provides a broad range of ecological niches and supports host‐specific fungal communities (Peay et al. 2013; Tedersoo et al. 2014). In our study, differences in plant community composition between Akure and Oba Hills Forest Reserves likely contributed to the site‐specific fungal assemblages. In addition, the warm, humid, and climatically stable conditions in tropical forests promote continuous fungal activity throughout the year (Egidi et al. 2019; Tedersoo and Bahram 2019). Both reserves experience mean annual temperatures of approximately 27°C and annual rainfall between 1350 and 1700 mm, conditions that are highly conducive to year‐round soil fungal activity. This interpretation is consistent with findings from East African rainforests, where mean annual temperature was identified as a stronger predictor of fungal community composition than bacterial community composition (Ren et al. 2024). Similarly, previous studies from tropical forest ecosystems in Africa and elsewhere have shown that climatic and edaphic conditions are major determinants of soil fungal community composition across spatial and temporal scales (Piepenbring et al. 2015; Olou et al. 2019; Ren et al. 2024; Solanki et al. 2024; Broeckhoven et al. 2025).

Finally, high litter production and pronounced soil heterogeneity in tropical forests create diverse substrates and microhabitats that facilitate the coexistence of multiple fungal lineages (Talbot et al. 2014; Huang et al. 2025). In both study sites, the undisturbed forests with their dense canopies and diverse tree species likely provided substantial and varied litter inputs that supported the observed saprotrophic fungal communities. Long‐term ecological stability may also promote the accumulation and specialization of fungal taxa in these ecosystems (Peay et al. 2016; Bahram et al. 2018). Although both forest reserves have experienced varying levels of anthropogenic disturbance, protected areas such as the Queen's plot in Akure Forest Reserve have maintained relatively stable conditions due to their designation as Strict Nature Reserves by the Nigerian government. These areas were established to preserve biodiversity and serve as reference ecosystems for scientific research and environmental monitoring.

### 4.2 Land‐Use Effects

Land‐use change had relatively limited effects on AMF communities but more pronounced effects on total fungal communities, particularly saprotrophic and plant pathogenic fungi. AMF are obligate biotrophs that depend on living plant roots and interconnected hyphal networks for survival (Smith and Read 2008). In the studied croplands, perennial agroforestry crops such as cocoa, banana, and plantain maintain continuous root systems and partial canopy cover. These conditions likely help preserve AMF extraradical hyphal networks following land‐use conversion, thereby buffering AMF communities against disturbance.

In contrast, saprotrophic fungal communities showed higher diversity in undisturbed forests than in croplands. This finding is in line with previous studies showing that forest disturbance reduces saprotrophic fungal activity due to reduced litter inputs and altered microclimatic conditions that constrain decomposition processes (Paudel et al. 2015; Gautam and Mandal 2017; Krashevska et al. 2018). Unlike AMF, saprotrophic fungi do not rely on persistent host‐associated hyphal networks and must recolonize disturbed habitats from spores or mycelial fragments, which makes them more sensitive to land‐use change. The high relative abundance of *Aspergillus* and *Penicillum* is noteworthy. Both genera include fast‐growing ant tolerant species that tend to proliferate under low water availability and high temperatures. Although, soil samples were air‐dried at room temperature, and air‐drying may have contributed to the enrichment of these saprotrophic fungi, their consistent dominance across all land‐use types, in comparison to other genera, likely suggests their ecological significance in the study sites. Previous studies have also reported *Aspergillus* and *Penicillium* as predominant soil saprotrophs in tropical ecosystems, mainly due to their ability to decompose complex organic substances and tolerate environmental stress (Jena et al. 2015; Do Nascimento Barbosa et al. 2016; Egidi et al. 2019).

In contrast, plant pathogenic fungi increased in diversity and showed a shift in community composition following conversion to cropland. Agricultural systems can promote pathogen persistence through continuous host availability and reduced microbial competition, which may favor the proliferation of plant pathogenic fungi (Monkai et al. 2022). Land‐use intensity has been shown to significantly influence soil physicochemical properties and fungal communities in forest ecosystems (Iacono et al. 2025; Leiririo et al. 2026). In our study, most soil chemical parameters were higher in undisturbed forests than in disturbed forests and croplands. This likely reflects greater stability of these ecosystems and the absence of anthropogenic disturbances such as tillage, fertilization and removal of native vegetation cover.

Overall, effects of land‐use were more pronounced on community composition than on diversity, which is in line with previous studies that have found that conversion of tropical forests to croplands does not necessarily lead to a severe reduction in fungal biodiversity but it does lead to a pronounced shift in fungal community composition (Brinkmann et al. 2019). However, these local shifts in community composition can globally lead to “biotic homogenization” (Peng et al. 2024). The biotic homogenization hypothesis poses that disturbance filters out specialists in favor of wide‐ranging generalists (McKinney and Lockwood 1999). This pattern is also supported by our indicator species analysis, which revealed that undisturbed forests harbored the highest number of unique OTUs. It indicates that while generalist fungi are resilient to land‐use change, specialist lineages restricted to native forest habitats are being lost. This process reduces the functional redundancy of the soil ecosystem even if species richness remains stable, highlighting the critical importance of conserving undisturbed forest habitats to maintain the full spectrum of below‐ground biodiversity (Birkhofer et al. 2018).

### 4.3 Differences Between Forest Reserves

Study site explained more variation in total soil fungal communities and major ecological groups than land‐use type, suggesting that local habitat conditions exert stronger control over fungal community composition than differences in broad land‐use categories. This strong site specificity could reflect differences in edaphic conditions between the forest reserves. Previous research has shown that factors such as pH, phosphorus, C:N ratio, total organic carbon, and nutrient availability can act as major environmental filters for fungal communities, including AMF (Davison et al. 2015; Olanipon et al. 2024; Rodríguez‐Echeverría et al. 2017), and they may therefore contribute to the differences observed between the study sites. The C:N ratio is widely used as an indicator of ongoing decomposition processes, nutrient cycling, and fungal activity (Mooshammer et al. 2014). In this study, higher C:N ratios in disturbed forests of Akure Forest Reserve suggest relatively slower decomposition, while lower C:N ratios in disturbed forests of Oba Hills Forest Reserve suggest faster decomposition rates and high saprotrophic fungal richness. Furthermore, due to more intense anthropogenic activities in Oba Hills Forest Reserve, the vegetation composition of the two reserves likely selects for different host‐associated fungal partners via variation in root exudates and litter quality (Peay et al. 2016).

Soil chemical properties were more stable in undisturbed forests of both reserves. For example, near‐neutral pH and higher total organic carbon in undisturbed forests likely contributed to the higher fungal diversity observed. Soil pH is a key predictor of microbial community structure and fungal communities tend to shift across pH gradients (Rousk et al. 2010; Leiririo et al. 2026), while organic carbon provides an important energy source, especially for saprotrophic fungi. The redundancy analysis corroborates this by identifying soil physicochemical properties as significant drivers of fungal community composition. The stronger response of the total fungal community to soil variables suggests that edaphic factors play a major role in shaping the broader fungal assemblage than the AMF community. Given the weaker effect of soil properties on AMF communities, additional factors such as host plant communities and spore dispersal processes likely contribute more to AMF community assemblage than soil properties alone.

Soil texture may also have contributed to differences in fungal community composition in both reserves. The sandy nature of soils in disturbed forests of both sites is coarse and has lower water and nutrient retention capacity, making them more vulnerable to erosion and runoff than clay‐rich soils (Holz and Augustin 2021). This can reduce nutrient availability and constrain fungal activity. In contrast, higher silt content in the undisturbed forests of Akure Forest Reserve may enhance water and nutrient retention, which in turn promotes fungal proliferation. The marked turnover in fungal community composition between sites implies that preserving a single forest reserve is unlikely to conserve the full regional fungal biodiversity. Instead, conservation strategies should incorporate a network of protected forest habitats to preserve site‐specific microbial assemblages and maintain high regional fungal diversity. Given the exceptionally high diversity of tropical forest fungi, the two forest reserves examined in this study will most likely also not be sufficient to capture the full extent of belowground diversity in the region, thus further emphasizing the importance of conserving multiple forest ecosystems.

### 4.4 AMF Communities

Arbuscular mycorrhizal fungi play an important role in tropical ecosystems as tropical tree species often associate with AMF (Van Der Heijden et al. 2015; Spatafora et al. 2017). Previous research in Nigeria has shown that tropical trees associate with multiple AMF taxa, and many AMF display broad host ranges and may function as hubs in belowground interaction networks (Olanipon et al. 2024). Consistent with these patterns, we found a large diversity of AMF in our study, with most taxa belonging to *Glomus*, *Paraglomus*, *Diversispora*, and *Acaulospora*, with site‐specific occurrences of *Claroideoglomus* and *Dentiscutata*. These findings are in line with other studies from Africa (Menge 2023; Touré et al. 2023) and elsewhere, which consistently identify *Glomus* as the dominant AMF genus (Boeraeve et al. 2019; Haug et al. 2021; Ji et al. 2022; Mwangi et al. 2026; Olanipon et al. 2024; Sheldrake et al. 2017; Suárez et al. 2023; Waymouth et al. 2022). Soil pH in the studied sites fell within ranges generally considered suitable for AMF, suggesting that pH was not a major limiting factor for AMF community composition. In contrast, the higher CEC and TOC in undisturbed forests may have enhanced nutrient availability and supported more stable AMF hyphal networks.

Contrary to our initial hypothesis, AMF richness and composition were not significantly influenced by land‐use type, but differed between the two sites. This suggests that the level of disturbance was not large enough to drive taxa locally to extinction and that most of them were able to persist on the remaining plant species. Unlike intensive monocultures, where fungal diversity often declines sharply (Wang et al. 2018), the croplands in our study sites (Akure and Oba Hills) are characterized by mixed systems of agroforestry and perennial crops (e.g., *Theobroma cacao* , *Musa* spp.) that maintain permanent root networks and canopy cover. These conditions likely preserve AMF hyphal networks and buffer the negative impacts of deforestation on soil microbial communities better than mechanized agriculture (Morris et al. 2013; Simons et al. 2017; Sepp et al. 2018).

## 5 Conclusion

This study evaluated the effects of land‐use changes on soil fungal, arbuscular mycorrhizal, saprotrophic, and plant pathogenic fungal communities in the Akure and Oba Hills Forest Reserves in Nigeria. In line with our hypotheses, land‐use change significantly affected soil fungal communities, especially saprotrophic and plant pathogenic fungal communities. On the other hand, land‐use type had minimal, non‐significant effects on the arbuscular mycorrhizal fungal communities, likely due to the perennial nature of local agroforestry systems that maintain continuous root networks. Although disturbance intensity was not explicitly quantified, strong compositional turnover between the two forest reserves highlights that soil fungal biodiversity is spatially structured and site‐specific. Therefore, effective conservation strategies must prioritize the protection of multiple, distinct forest fragments rather than a single protected area to maintain site‐specific microbial assemblages and regional fungal diversity. In parallel, promoting agroforestry practices that maintain root continuity may help sustain soil health in human‐modified landscapes.

## Funding

This research was supported with an Underground Explorers grant from the Society for the Protection of Underground Networks (SPUN) awarded to Damilola Olanipon (UE2022‐23). Margaux Boeraeve is supported by a Research Foundation Flanders fellowship (1273125N).

## Conflicts of Interest

The authors declare no conflicts of interest.

## Supporting information


**Figure S1:** Sampling Grid (Source: https://www.spun.earth/expeditions/sampling‐protocol).
**Figure S2:** Rarefaction curves for all samples in the ITS2 dataset (total fungal communities).
**Figure S3:** Rarefaction curves for all samples in the SSU dataset (AMF communities).
**Figure S4:** NMDS ordinations based on Bray‐Curtis distances for all fungi, plant pathogenic fungi and saprotrophic fungi and a PCoA ordination for arbuscular mycorrhizal fungi (AMF). The stress value of an NMDS ordination for AMF was too high (stress = 0.248), hence the inclusion of a PCoA instead.

## Data Availability

The sequence datasets presented in this study can be found in the online repository ‐ https://www.ncbi.nlm.nih.gov/sra/, with accession number – PRJNA1396320.
