# Supplementary material for: Land‐Use Shapes Soil Fungal Communities and Functional Guilds in Tropical Forest Ecosystems
Source: Plant Environ Interact. 2026 Aug 4;7(4):e70193. doi: 10.1002/pei3.70193 (PMC13438191; doi:10.1002/pei3.70193)
Supplement: Supplementary file 1 [file PEI3-7-e70193-s001.docx]

**Supporting Information**

**Fig. S1** Sampling Grid (Source: <https://www.spun.earth/expeditions/sampling-protocol>)

**Fig. S2** Rarefaction curves for all samples in the ITS2 dataset (total fungal communities).

**Fig. S3** Rarefaction curves for all samples in the SSU dataset (AMF communities)

**Fig. S4** NMDS ordinations based on Bray-Curtis distances for all fungi, plant pathogenic fungi and saprotrophic fungi and a PCoA ordination for arbuscular mycorrhizal fungi (AMF). The stress value of an NMDS ordination for AMF was too high (stress = 0.248), hence the inclusion of a PCoA instead.
